# Body mass index associations between mother and offspring from birth to age 18: the Fels Longitudinal Study

**DOI:** 10.1002/osp4.90

**Published:** 2017-04-26

**Authors:** S. Swanton, A. C. Choh, M. Lee, L. L. Laubach, J. K. Linderman, S. A. Czerwinski, M. J. Peterson

**Affiliations:** ^1^ Department of Health and Sport Science, School of Education and Health Sciences University of Dayton Dayton USA; ^2^ Division of Epidemiology and Biostatistics, Department of Population and Public Health Sciences, Boonshoft School of Medicine Wright State University Dayton USA; ^3^ Department of Epidemiology, Human Genetics and Environmental Sciences, School of Public Health University of Texas Health Science Center (UTHealth) Brownsville USA

**Keywords:** body mass index (BMI), human growth and development, obesity

## Abstract

**Background:**

Parental obesity is a known determinant of childhood obesity. Previous research has shown a strong maternal influence on body mass index (BMI) during infancy and early childhood.

**Objectives:**

The purpose of this research was to investigate the BMI associations between mother and offspring from birth to age 18 years.

**Methods:**

Participants were selected from the Fels Longitudinal Study. The current study sample includes 427 (215 mother/son and 212 mother/daughter) mother/child pairs. These pairs are repeatedly measured at multiple age groups in children, resulting in a total of 6,263 (3,215 mother/son, 3,048 mother/daughter) observations for data analysis. Inclusion criteria were children with measured height and weight for BMI collected at ages 0 to 18 years and their mother with BMI data. Maternal influences of BMI on offspring BMI from birth to early adulthood were analyzed by Spearman correlations and linear regression analyses.

**Results:**

Mother/son BMI correlations became statistically significant (*p* ≤ 0.05) at age 5–6 years and were significant through puberty and into early adulthood at age 18 years. Mother/daughter correlations became significant at age 1.5 years and also continued through adolescence, puberty and early adulthood at age 18 years. Associations persisted after the study sample was grouped into life stages and adjusted for decade of birth and parity.

**Conclusions:**

The mother/daughter relationship was more strongly correlated than the mother/son relationship and also became statistically significant at an earlier age than boys.

## Introduction

Approximately two out of three Americans adults and one in three children are classified as overweight or obese 1. Chronic diseases such as metabolic syndrome, cardiovascular disease and hypertension, along with mortality in adulthood, are serious health issues that arise from being overweight and obese in childhood 2, 3, 4, 5, 6, 7, 8, 9, 10, 11. The appearance of chronic diseases during childhood, as a consequence of obesity, is also a major public health concern in the USA 11. Ogden *et al*. noted that while there has been no significant increase in the prevalence of the obesity epidemic in children or adults between the years 2003–2004 and 2011–2012, obesity rates are still high and remain a major public health concern 1.

Because parents and children share both genes and a familial environment, parental obesity is a predominant risk factor for childhood obesity 12, 13, 14. A study in 2003 by Magarey *et al*. compared body mass index (BMI) in 155 children and their parents. They found that as children aged, the prevalence of overweight/obesity increased and continued at age 20 years 14. They also found son's BMIs were significantly correlated to their maternal BMI at every age and to paternal BMI at every age except at 20 years. Similarly, the daughter's BMI was significantly correlated to maternal BMI beginning at age eight, and paternal BMI beginning at age four 14.

A recent study by Linabery *et al*. examined the influence of maternal obesity on infant's height and weight from birth to 3 years of age and compared the findings to infant BMI growth curves. They reported that at birth and later into infancy, there is a greater maternal than paternal influence on infant BMI 15. Additionally, in a longitudinal study examining anthropometric relationships between parents and children at different pubertal stages, maternal BMI and weight correlations were significant at birth and maternal waist‐to‐hip ratio, and skin‐fold measurements were significant at puberty 13.

Overall, maternal anthropometric measurements have been found to have significant influences on children in two important stages in their life, during the neonatal period and puberty 13. These findings support the idea that familial factors, such as parents' body composition, are correlated with offspring body composition at various ages and stages throughout childhood. Our previous research conducted using the Fels Longitudinal Study provided some evidence in significant parental and offspring correlations in BMI over a small age range from birth to 3 years 15. The knowledge of whether these associations hold during other periods of growth such as early adulthood is lacking.

The purpose of this study is to investigate the BMI associations between mother and offspring through growth; beginning from birth through age 18, determining if, at what age, children resemble their mother with regard to BMI. It is hypothesized that there will be a statistically significant relationship between mother and offspring through multiple ages and growth stages independent of secular or birth order influences. Further, we hypothesize that there will be a stronger correlation between mothers and daughters, than mothers and sons, due to the same sex relationship.

## Methods

### Study sample

Information regarding the Fels Longitudinal Study has been previously published in detail 16. Briefly, the study began in 1929, where researchers observed the growth, maturation and body composition of citizens residing in the Dayton/Yellow Springs, Ohio area. Individuals were enrolled in this study *in utero*, along with pre‐existing family members and followed throughout their lifespan. Participants were not selected with regard to any known factors associated with health concerns, diseases or body composition. All study procedures were approved by the Institutional Review Board at Wright State University. All adult participants provided informed consent prior to participation. Parents of children under 18 years of age provided consent for their children, and their children between ages 7 and 18 years provided assent for the participation.

Mother–child pairs who both had height, weight were first identified. Inclusion criteria for this study required that mothers had data at an age range close to 35 ± 5 years (30‐ to 40‐years‐old). The birth years of the mothers ranged from 1892 to 1983. Children had to have BMI data, such as height and weight, at least once between ages 0 and 18 years. The birth years of the children ranged from 1928 to 2012.

In order to more accurately compare BMI associations over multiple age groups and childhood stages, data for children were binned into target age groups. For each half‐year target age category from 0 to 3 years, the age interval was 6 months (target age ± 3 months), while each full‐year age category from 4 to 18 years was a full year interval (target age ± 6 months). The half‐year target age groups were chosen in order to focus on the high velocity of growth that occurs in early developmental ages between birth and age three 17. The study sample was later grouped into life stages for further data analysis. These life stages included infancy (0–2 years), early childhood (2.5–6 years), school age (7–10 years), early adolescence (11–15 years) and late adolescence (16–18 years). Boys and girls were each grouped into these categories separately.

There are a total of 1,612 children aged 0–18 years in the current Fels data set. Based on the inclusion criteria, the final study sample included 427 (215 mother/son and 212 mother/daughter) mother/child pairs. Aside from a very small portion with no height or weight data, the excluded children are almost exclusively those whose mother's age is lower than 30 or greater than 40 years. The included pairs are repeated across multiple age groups, resulting in a total of 6,263 (3,215 mother/son and 3,048 mother/daughter) observations for data analysis. Of the 427 mother–child pairs, 86 pairs were included in all target age groups in children.

### Anthropometrics

Anthropometric measurements (height and weight) were taken at regular, scheduled visits using standardized methods 18. Visit schedules depend on the age and sex of the participant. Measurements during infancy occur at 0, 1, 3, 6, 9 and 12 months. After age 1, children are assessed semi‐annually until age 18, then every 2 years following during adulthood. As previously mentioned, these data were binned into target age groups.

In order to increase accuracy and reliability of the measurements, data measurements need to be within 0.2 kg for weight and 1.0 cm for height. Infants' recumbent lengths were measured to the nearest 0.1 cm using an infant length board until 2 years of age, when they were old enough to stand for vertical height using a Holtain stadiometer (Seritex, Carlstadt, NJ). Weight (kg) was measured to the nearest 0.01 kg without heavy clothing. Infants had all measurements taken wearing only a diaper. Adult measurements were obtained in a similar fashion during scheduled data collection visits where vertical height was taken wearing no shoes, and weight was measured wearing light clothing. BMI was calculated as weight/height (kg m^−2^) using either recumbent length for children until 2 years of age or standing height for all other ages.

### Statistical analysis

spss Statistics 23.0 (IBM Corporation, USA), Microsoft Excel and sas (SAS Institute Inc., Cary, NC) were used for statistical analysis. Sex‐stratified analyses were performed for all analyses. Descriptive statistics were reported using mean ± standard deviation (SD). Univariate statistics were used to compare the BMIs of mothers and offspring in order to determine the shape of the distribution. Because data were not normally distributed, Spearman correlation coefficient analyses were used to conduct non‐parametric testing of the degree of association between the mothers and offspring (*p* ≤ 0.05). After performing Spearman correlations, multiple linear regression analyses were conducted for each sex and target age group to examine the influence of mother's BMI on child's BMI. Covariates included in the multiple regressions included mother's birth year to control for secular trends. Benjamini–Hochberg multiple comparisons tests were used for all analyses 19. The nominal significance level for statistical testing was set at *α* = 0.05.

The sample was then grouped into life stages based on the child's age, as explained previously. Repeated measures linear regressions were then conducted for both boys and girls life stage groupings. Covariates included were maternal BMI, maternal birth year and/or material parity (i.e. offspring's birth order).

## Results

The means ± SD, and ranges (minimum, maximum) for BMI values of offspring are depicted for each target age category (Table 1). Both boys and girls had a similar mean BMI of 13.3 kg m^−2^ (SD = 1.5 and 1.3, respectively) at birth. Mean BMI of mothers in this study sample was 23.8 kg m^−2^. Generally, BMI increased as age increased in both mother/son and mother/daughter pairings. Both relationships also demonstrated heteroscedasticity as age increased (Table 1).

**Table 1:** Descriptive statistics for boy and girls in mother/son and mother/daughter pairs

Target age	Son		Daughter	
	*N*	BMI mean + SD (min, max)	*N*	BMI mean + SD (min, max)
0	177	13.3 ± 1.5 (8.0, 18.5)	178	13.3 ± 1.3 (8.9, 17.2)
0.5	153	17.3 ± 1.4 (13.7, 20.9)	154	16.8 ± 1.5 (13.3, 21.2)
1	155	17.4 ± 1.2 (14.8, 20.6)	155	17.0 ± 1.3 (14.2, 20.2)
1.5	147	16.6 ± 1.1 (14.2, 19.2)	146	16.4 ± 1.3 (12.9, 19.3)
2	144	16.3 ± 2.0 (13.8, 21.0)	137	16.0 ± 1.2 (13.1, 18.6)
2.5	136	16.0 ± 1.2 (13.7, 22.7)	141	15.7 ± 1.2 (11.9, 18.5)
3	151	15.9 ± 1.1 (13.2, 19.7)	145	15.7 ± 1.2 (12.1, 18.8)
4	154	15.7 ± 1.2 (13.1, 22.1)	156	15.6 ± 1.2 (12.8, 19.1)
5	157	15.6 ± 1.3 (13.1, 21.6)	144	15.5 ± 1.4 (12.3, 20.1)
6	148	15.6 ± 1.5 (12.8, 23.3)	143	15.5 ± 1.6 (12.3, 21.9)
7	159	15.8 ± 1.8 (12.8, 27.3)	146	15.9 ± 2.0 (12.1, 25.0)
8	156	16.1 ± 2.2 (12.9, 30.0)	144	16.2 ± 2.3 (12.6, 24.2)
9	148	16.7 ± 2.7 (13.0, 30.9)	142	17.0 ± 2.8 (12.5, 25.8)
10	155	17.3 ± 3.0 (13.0, 33.6)	152	17.7 ± 3.2 (12.3, 27.4)
11	150	18.0 ± 3.3 (13.3, 34.7)	147	18.5 ± 3.5 (13.0, 30.2)
12	143	18.5 ± 3.4 (14.0, 36.0)	138	19.2 ± 3.7 (12.5, 29.7)
13	145	19.1 ± 3.4 (14.0, 38.3)	134	20.0 ± 3.8 (14.0, 33.4)
14	148	20.1 ± 3.9 (14.9, 40.0)	134	20.9 ± 4.0 (14.7, 32.9)
15	141	20.9 ± 3.8 (15.4, 38.2)	115	21.3 ± 3.9 (15.3, 36.5)
16	137	21.4 ± 4.1 (15.3, 43.7)	118	21.3 ± 3.9 (15.3, 36.5)
17	137	21.9 ± 3.2 (15.4, 40.7)	113	21.8 ± 4.0 (16.1, 36.3)
18	74	21.6 ± 2.8 (17.0, 31.3)	66	21.6 ± 3.6 (16.4, 35.9)

BMI, body mass index; *N*, sample size; SD, standard deviation.

**Figure 1:**
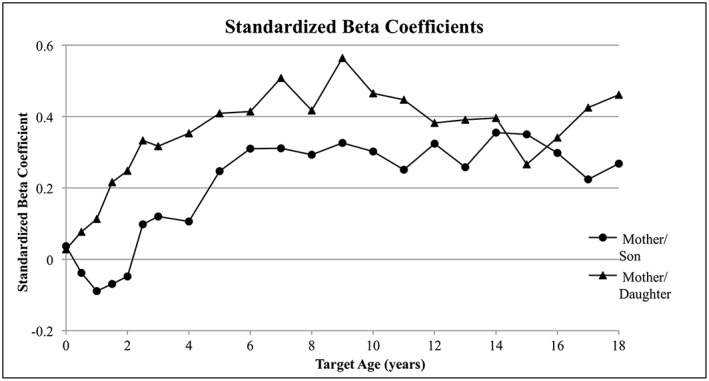
Adjusted standardized beta coefficients for the relationship between mother and child BMI across all target ages.

The mother/son relationship becomes significant (*p* ≤ 0.05) at target age 6 and remains constantly significant through age 18 (Table 2). The mother/daughter correlation starts to become significant (*p* ≤ 0.05) at target age 1.5 and remains significant through age 18, peaking at age 9 years (Table 3). Overall, the mother/daughter relationship has stronger, and earlier, significant correlations compared with the mother/son relationship (Figure 1). After grouping the study sample into life stages and controlling for decade of birth and parity, the associations observed in the correlations analyses remained for the most part unchanged for mothers/sons (Tables 4) and mothers/daughters (Table 5). The one exception was the significant mother/infant daughter association that was observed. However, it is plausible that this significant association was driven primarily by later infancy correlations already observed in bivariate correlations. This highlights the importance and novelty of examining these relationships in half‐year groupings, as opposed to life stages, which does not allow for finite examination of infancy. Several other interesting findings were noted in the regression analyses of life stages. Infant boys in the earlier decades had a higher BMI compared with those in the more recent decades. There was no significant parity effect found in any regression models and therefore did not have an effect on the BMI correlations in this sample. In regard to decade of birth, we observed a negative decade effect in infancy and early childhood life stages in mother/son and mother/daughter models, meaning that children born in earlier 1900s decades had a higher BMI in these early life stages. In the other three life stages (school age, early adolescence and late adolescence), higher BMI values in children were associated with later decades of birth.

**Table 2:** Relationships between maternal BMI and son's BMI at each target age

Target age	*N*	Spearman correlation (rho)	Standardized *β* coefficient
0	177	0.047	0.037
0.5	153	−0.041	−0.038
1	155	−0.072	−0.089
1.5	147	−0.040	−0.069
2	144	−0.038	−0.048
2.5	136	0.056	0.098
3	151	0.075	0.120
4	154	0.055	0.106
5	157	0.171	0.247**
6	148	0.206*	0.310**
7	159	0.207*	0.311**
8	156	0.165	0.293**
9	148	0.221*	0.326**
10	155	0.216*	0.302**
11	150	0.159*	0.251**
12	143	0.220*	0.324**
13	145	0.226*	0.258**
14	148	0.260*	0.355**
15	141	0.273*	0.350**
16	137	0.266*	0.298**
17	137	0.263*	0.224**
18	74	0.264*	0.268**

Standardized *β* coefficients were obtained from linear regression models adjusting for maternal birth year.

* Significance at *p* ≤ 0.05.

** Significance at adjusted *p* ≤ 0.05.

BMI, body mass index; *N*, sample size.

**Table 3:** Relationship between maternal BMI and daughter's BMI at each target age

Target age	N	Spearman correlation (rho)	Standardized *β* coefficient
0	178	0.025	0.028
0.5	154	−0.032	0.077
1	155	0.110	0.113
1.5	146	0.176*	0.216**
2	137	0.173	0.248**
2.5	141	0.248*	0.333**
3	145	0.264*	0.317**
4	156	0.338*	0.353**
5	144	0.339*	0.409**
6	143	0.333*	0.414**
7	146	0.390*	0.508**
8	144	0.380*	0.417**
9	142	0.424*	0.564**
10	152	0.402*	0.465**
11	147	0.382*	0.447**
12	138	0.389*	0.382**
13	134	0.366*	0.391**
14	134	0.374*	0.396**
15	115	0.345*	0.266**
16	118	0.272*	0.341**
17	113	0.272*	0.425**
18	66	0.269*	0.461**

Standardized *β* coefficients were obtained from linear regression models adjusting for maternal birth year.

* Significance at *p* ≤ 0.05.

** Significance at adjusted *p* ≤ 0.05.

BMI, body mass index; *N*, sample size.

**Table 4:** Mother/son linear regression models adjusting for decade of birth and parity

Life stage	*N*	*Mother's BMI	*Decade of birth	*Parity
Infancy	197	−0.006	−0.01	0.02
		(0.75)	(0.001)	(0.70)
Early childhood	183	0.07	−0.004	0.02
		(0.001)	(0.25)	(0.70)
School age	181	0.17	0.01	−0.08
		(<0.0001)	(0.04)	(0.51)
Early adolescence	176	0.22	0.03	−0.06
		(<0.0001)	(0.003)	(0.76)
Late adolescence	156	0.28	0.01	−0.02
		(<0.0001)	(0.25)	(0.93)

* Values are reported as standardized *β* coefficients (significance value).

BMI, body mass index; *N*, sample size.

**Table 5:** Mother/daughter linear regression models adjusting for decade of birth and parity

Life stage	*N*	*Mothers BMI	*Decade of birth	*Parity
Infancy	196	0.05	−0.01	−0.06
		(0.01)	(0.002)	(0.48)
Early childhood	185	0.09	−0.00	−0.15
		(<0.0001)	(0.36)	(0.09)
School age	170	0.18	0.03	−0.12
		(<0.0001)	(<0.0001)	(0.53)
Early adolescence	165	0.215	0.06	−0.12
		(<0..0001)	(<0.0001)	(0.65)
Late adolescence	133	0.30	0.05	−0.44
		(<0.0001)	(<0.0001)	(0.12)

* Values are reported as standardized *β* coefficients (significance value).

BMI, body mass index; *N*, sample size.

## Discussion

Utilizing over 90 years of data, the current study examined the association of maternal BMI to her offspring's BMI through the first 18 years of their lives. A significant correlation between mother and child BMI has been identified. Daughters BMI became significantly associated with the maternal BMI as early as age 1.5 years, while the sons' BMIs with the maternal BMI began at age 5 years, and through age 18 for both genders.

Generally, associations between offspring BMI and maternal BMI at each target age group were stronger in daughters than sons. The correlation coefficients were generally higher in the mother/daughter relationship, beginning in early childhood and persisting through adolescence, and into early adulthood. Among sons, mother/son relationship only became statistically significant in adolescence and proceeded through puberty, and into early adulthood. Both of these relationships remained significant after controlling for secular trends and adjusting for the Benjamini–Hochberg correction to control for multiple comparisons across age and sex.

Previous research demonstrated that obesity in early adulthood could be predicted from childhood and parental obesity 13. It was concluded that children whose both parents are classified as overweight are at a higher risk for becoming overweight than those who have either one or no parent overweight 14. Body weight of parents has a direct influence on a child's body weight from birth, into developmental stages, and through early adulthood. Safer *et al*. reported significant correlations between both maternal and paternal BMI and their children's BMI at the age of 7, whereas the current study found statistical significance beginning as early as 1.5 years for daughters with maternal BMI 20. Our results differ somewhat, however, from the Fleurbaix–Laventie Ville Sante Study that examined various anthropometric parameters in parents and children. In this longitudinal study, BMI and weight at birth were highly correlated with maternal anthropometry 21. The study also reported that maternal adiposity affects offspring's adiposity in the early ages of life. While the findings are similar to our current study with regard to maternal influence on offspring during childhood, in our sample, there were no maternal influences at birth.

The maternal relationship has been established as more influential on offspring BMI 15. Linabery *et al*. previously reported that maternal BMI has a significantly stronger association on BMI in infancy and early childhood 15. Our study supports these previous findings of association from infancy and early childhood and extends the relationship into early adulthood. Because previous research has demonstrated that the paternal relationship is not as significant as maternal, the current researchers did not explore the association between fathers and offspring.

The current study is the only known anthropometric study that examines the association of maternal BMI to the BMI of her offspring throughout an 18‐year span starting from birth. This unique study provides many insights to the extent of maternal influence on their children's BMI. The significant findings can include both shared genetic and shared environmental affects; however, this study does not distinguish between them. Understanding this relationship could provide assistance for targeting behavioural interventions at specific stages of childhood development that would be most effective in addressing the health concerns associated with overweight and obesity.

Targeted interventions at certain life stages could be useful in addressing obesity trends between mother and offspring. Although these interventions may take place at different times for sons versus daughters, they are equally as important. For example, because the BMI association between mother and daughter pairs becomes significant at the daughters age of 1.5 years, a lifestyle intervention or family‐based intervention at this time would be pertinent. Because the mother–son relationship became significant at the boys age of 6 years, this would be the critical age at which a lifestyle intervention would be most successful for both mother and son. The significant relationships give insight to the importance of having both mother and child partake in such lifestyle changes in order to prevent obesity or fat mass early 22. Examples of such lifestyle interventions could be an increase in physical activity along with incorporation of healthy eating habits.

This study exclusively includes European–American children residing in southwest Ohio and therefore may limit application to other ethnic groups in this region. The current sample is restricted to the mother–offspring correlation; hence, future research should include the father–offspring relationship. Another limitation to the present investigation is the comparison of BMI of mothers at a fixed age (35 ± 5 years) to her offspring at various ages. Because the Fels study began in 1929, the average BMI of the participants in this study sample does not reflect those of present day USA. Future research is needed to compare parent and child BMI at corresponding ages, as well as exploring these associations after separating out current genetic factors.

## Conflict of interest statement

No conflict of interest was declared.

## References

[1] Ogden CL , Carroll MD , Kit BK , Flegal KM . Prevalence of childhood and adult obesity in the United States, 2011–2012. JAMA 2014; 311: 806–814. 24570244 10.1001/jama.2014.732 PMC4770258

[2] Sabo RT , Yen MS , Daniels S , Sun SS . Associations between childhood body size, composition, blood pressure and adult cardiac structure: the Fels Longitudinal Study. PLoS One 2014; 9: e106333. 25191997 10.1371/journal.pone.0106333 PMC4156369

[3] Sun SS , Liang R , Huang TT , et al. Childhood obesity predicts adult metabolic syndrome: the Fels Longitudinal Study. J Pediatr 2008; 152: 191–200. 18206688 10.1016/j.jpeds.2007.07.055 PMC3988700

[4] Freedman DS , Khan LK , Dietz WH , Srinivasan SR , Berenson GS . Relationship of childhood obesity to coronary heart disease risk factors in adulthood: the Bogalusa Heart Study. Pediatrics 2001; 108: 712–718. 11533341 10.1542/peds.108.3.712

[5] Guo SS , Wu W , Chumlea WC , Roche AF . Predicting overweight and obesity in adulthood from body mass index values in childhood and adolescence. Am J Clin Nutr 2002; 76: 653–658. 12198014 10.1093/ajcn/76.3.653

[6] Wang Y , Beydoun MA . The obesity epidemic in the United States ‐ gender, age, socioeconomic, racial/ethnic, and geographic characteristics: a systematic review and meta‐regression analysis. Epidemiol Rev 2007; 29: 6–28. 17510091 10.1093/epirev/mxm007

[7] Siervogel RM , Wisemandle W , Maynard LM , Guo SS , Chumlea WC , Towne B . Lifetime overweight status in relation to serial changes in body composition and risk factors for cardiovascular disease: the Fels Longitudinal Study. Obes Res 2000; 8: 422–430. 11011908 10.1038/oby.2000.52

[8] Lawlor DA , Leon DA . Association of body mass index and obesity measured in early childhood with risk of coronary heart disease and stroke in middle age: findings from the Aberdeen children of the 1950s prospective cohort study. Circulation 2005; 111: 1891–1896. 15837941 10.1161/01.CIR.0000161798.45728.4D

[9] Must A . Morbidity and mortality associated with elevated body weight in children and adolescents. Am J Clin Nutr 1996; 63: 445S–447S. 8615339 10.1093/ajcn/63.3.445

[10] Must A , Strauss RS . Risks and consequences of childhood and adolescent obesity. Int J Obes Relat Metab Disord 1999; 23: S2–11. 10.1038/sj.ijo.0800852 10340798

[11] Freedman DS , Mei Z , Srinivasan SR , Berenson GS , Dietz WH . Cardiovascular risk factors and excess adiposity among overweight children and adolescents: the Bogalusa Heart Study. J Pediatr 2007; 150: 12–17. e12. 17188605 10.1016/j.jpeds.2006.08.042

[12] Sun SS , Deng X , Sabo RT , et al. Secular trends in body composition for children and young adults: the Fels Longitudinal Study. Am J Hum Biol 2012; 24: 506–514. 22410970 10.1002/ajhb.22256 PMC3372655

[13] Heude B , Kettaneh A , Rakotovao R , et al. Anthropometric relationships between parents and children throughout childhood: the Fleurbaix–Laventie Ville Sante Study. Int J Obes (Lond) 2005; 29: 1222–1229. 15795752 10.1038/sj.ijo.0802920

[14] Magarey AM , Daniels LA , Boulton TJ , Cockington RA . Predicting obesity in early adulthood from childhood and parental obesity. Int J Obes Relat Metab Disord 2003; 27: 505–513. 12664084 10.1038/sj.ijo.0802251

[15] Linabery AM , Nahhas RW , Johnson W , et al. Stronger influence of maternal than paternal obesity on infant and early childhood body mass index: the Fels Longitudinal Study. Pediatr Obes 2013; 8: 159–169. 23042783 10.1111/j.2047-6310.2012.00100.x PMC3765070

[16] Roche AF . Growth, Maturation and Body Composition: The Fels Longitudinal Study 1929–1991. Cambridge University Press: Cambridge: New York, NY, 1992.

[17] Choh AC , Lee M , Kent JW , et al. Gene‐by‐age effects on BMI from birth to adulthood: The Fels longitudinal study. Obesity 2014; 22: 875–881. 23794238 10.1002/oby.20517 PMC3883986

[18] Lohman TG , Roche AF , Martorell R . Anthropometric Standardization Reference Manual. Human Kinetics Publishers: Champaign, IL, 1988.

[19] Benjamini Y , Hochberg Y . Controlling the false discovery rate – a practical and powerful approach to multiple testing. J R Statist Soc B 1995; 57: 289–300.

[20] Safer DL , Agras WS , Bryson S , Hammer LD . Early body mass index and other anthropometric relationships between parents and children. Int J Obes Relat Metab Disord 2001; 25: 1532–1536. 11673777 10.1038/sj.ijo.0801786

[21] Botton J , Heude B , Maccario J , et al. Parental body size and early weight and height growth velocities in their offspring. Early Hum Dev 2010; 86: 445–450. 20580499 10.1016/j.earlhumdev.2010.06.001

[22] Meller F , Assuncao M , Schafer A , et al. The influence of birth order and number of siblings on adolescent body composition: evidence from a Brazilian birth cohort study. Brit J Nutr 2015; 114: 118–125. 26074279 10.1017/S0007114515001488 PMC4530600

